# Starting with Story, Not Symptom: Narrative Collapse and Cultural Repair in Appalachia

**DOI:** 10.13023/jah.0801.01

**Published:** 2026-04-01

**Authors:** Wayne Coombs

**Affiliations:** Independent

**Keywords:** Appalachia, Adverse Childhood Experiences (ACEs), coal and timber, Community resilience, Cultural repair, Epigenetics, Extraction economy, Intergenerational trauma, Narrative identity, Opioid crisis, Safety and coherence, Story circles, Trust and belonging

## Abstract

Appalachia’s opioid crisis reflects uneven burdens, especially in distressed coalfield counties, and it cannot be solved by symptom tracking alone. This commentary argues for a narrative shift, starting with story and cultural repair alongside clinical care. Narrative collapse is defined here as the loss of shared, dependable public meaning across groups, not the absence of storytelling in families, churches, and recovery circles. Trauma science suggests that chronic threat reshapes nervous systems across generations, making safety a prerequisite for renewed trust. Drawing on narrative identity research, the piece proposes peer story work as infrastructure that can reduce shame, support treatment engagement, and restore belonging.

## INTRODUCTION

In Appalachia and across rural America, institutions continue to search for answers to the opioid crisis. Millions of dollars are spent tracking symptoms, overdose rates, mortality, and addiction severity, but too little is invested in the cultural conditions that give rise to those symptoms. We must start with story, not symptom.

The 2019 report "Health Disparities Related to Opioid Misuse in Appalachia," published by East Tennessee State University (ETSU) and funded by the Appalachian Regional Commission (ARC), confirms what many in our communities already know: opioid misuse cannot be separated from the region’s structural, economic, and cultural history.^1^

The opioid crisis has hit unevenly across the 13-state region of Appalachia, and ARC’s work on diseases of despair shows that the heaviest burdens often fall on distressed Central Appalachian counties, including many in the coalfields.^2^,^3^ County-level research also links higher drug mortality with economic distress and mining dependence, a pattern that fits much of the coalfield geography.^4^,^5^

The misuse of opioids themselves is only the surface of the opioid crisis. Generations of economic dislocation, institutional betrayal, and social and cultural displacement have long been the underside of the crisis. Appalachia’s distrust in health care and the government at large are not unfamiliar stories, but similar distrust has been cultivated even between neighbors. We are dealing with the outcomes of environments where trauma has been normalized and resilience is expected without the necessary framework of support, safety, or recognition.

This distrust is passed on the same as genes for hair and eye color. The above report highlights the impact of Adverse Childhood Experiences (ACEs), acknowledging that trauma does not end when a generation passes. It becomes embedded in nervous systems, in stories, in the ways we raise our children. The developmental pathways that form in early childhood are not simply psychological; they’re social, cultural, and neurobiological. Trauma, particularly when it is chronic, complex, or intergenerational, reshapes the brain’s capacity to trust, to reflect, and to hope.^6^ Those pathways are shaped by history.

In Appalachia, we cannot separate today’s health crises from yesterday’s extraction. While coal, timber, and other resource industries removed wealth from the region, they removed also meaning, identity, and control. Generations of Appalachian families were caught in cycles of labor exploitation, environmental degradation, and corporate control over land, time, and even speech. These were not just economic harms. They were cultural traumas.

Coal mining, and the related blasting of mountains, posed a threat to culture as well as geography. The ensuing destruction of community similarly atomized families and deteriorated independence by causing community reliance upon institutions that did not value or invest in their long-term well-being. The result was economic depression as well as narrative collapse: people unsure of where they belonged, what they were worth, or what future they could hope for. Narrative collapse implies the disintegration of shared stories that once gave meaning, cohesion, and purpose to community life. This does not mean people stopped telling stories in families, churches, and recovery groups; it means those stories no longer connect across groups into a common public account that can support cooperation and belonging. When markets and governments fail communities through betrayal, abandonment, and upheaval, shared stories fray, and individual identity often frays with them.

If we want recovery, we have to start there. Not only with clinical care or economic development, but with the deeper work of cultural repair. That means giving a name to the harm. We have to be honest about what happened in Appalachia, not because we want to dwell in grievance, but because healing requires coherence. The body cannot heal from what the mind refuses to name.

We begin life shaped by the field around us. For most, we become reproducers of both biology and culture, two things which are intricately woven together. In too many Appalachian communities, the field itself has been damaged. Vigilance and fragmentation are passed on as a result of this damaged field, rather than trust and coherence. One can see this in the way a grandparent’s fear becomes a parent’s vigilance, which becomes a child’s anxiety.

This is not abstract. Work in cultural trauma and epigenetics, including research by Rachel Yehuda and others, suggests how trauma travels through biology and narrative alike. Epigenetics refers to how environmental stressors, like poverty, violence, or neglect, can alter gene expression without changing the DNA itself. These changes can influence how future generations respond to stress, even in the absence of the original trauma.^7^ This is not a claim that Appalachian culture is deficient or to blame, but rather a claim about what chronic threat, deprivation, and institutional betrayal do to bodies and relationships over time. The nervous system remembers and adapts, often in ways that served survival in the past but impair thriving in the present. A body trained for threat is not a body that rests easily. A community trained for betrayal is not one that trusts easily.

Psychiatrist Judith Herman emphasizes that trauma recovery begins not with revisiting the wound, but with creating physiological, emotional, and relational safety. Without safety, no story can truly be heard.^8^ Porges describes how bodies read cues of safety and danger through neuroception long before people can explain what they are sensing, and that shapes whether they can stay present with others.^9^ Yet the story itself is part of the process. You can’t rewrite the story until the nervous system is ready to hear it. But often, the right story, told in a safe enough space, is what makes the nervous system ready.

The ETSU report ends by calling for community resilience, intergenerational healing, and trauma-informed, place-aware care. These are not small asks, but they point in the right direction. The prescription is more holistic than medical; tending to generational trauma must happen in a community, as a community, through processes that foster the reintegration of story.

As psychologist Dan McAdams has argued, identity is formed in narrative.^10^ Kate McLean reinforces the idea that narrative is not only how we describe who we are, but rather is part of how we become who we are.^11^ Longitudinal work by Adler and colleagues links how people narrate agency, connection, and change with later mental health trajectories, which supports the claim that story is tied to well-being.^12^ In health care, narrative medicine argues that care improves when clinicians learn to hear and act on people’s lived accounts, and narrative approaches can reduce stigma in opioid use disorder care.^13^,^14^ These insights matter. In regions like Appalachia, where economic systems have collapsed and trauma has been inherited, rebuilding story is how we begin to rebuild self, culture, and connection.

The work of recovery in Appalachia must begin with building cultural trust. In many coalfield places, peer story work sometimes does what clinics cannot do well. It creates felt safety and belonging that make treatment usable, and it gives people a way back after a missed appointment or relapse by reconnecting local people, memory, and meaning. Story circles, shared history, and communal reflection form an infrastructure. They are what allow a fragmented culture to become coherent again.

If the question is “Yes, but how?”, start by connecting narrative-minded scholars with community organizers and existing recovery groups. Choose a trusted local place, keep the circle small enough to be safe, listen in turn, and turn what people say into a few commitments that are small enough to keep. Over time, those kept commitments become the beginnings of shared meaning again.

What if Appalachia’s recovery starts not in a clinic, but in a circle? Not with a diagnosis, but with a story? Let’s build systems that grow trust the way trauma grew distrust.

We start with story. And we grow from there.
